# Supplementary material for: Unlimited in vitro expansion of adult bi-potent pancreas progenitors through the Lgr5/R-spondin axis
Source: EMBO J. 2013 Sep 17;32(20):2708–21. doi: 10.1038/emboj.2013.204 (PMC3801438; doi:10.1038/emboj.2013.204)
Supplement: Supplementary Information [file emboj2013204s1.pdf]

PDL-WT

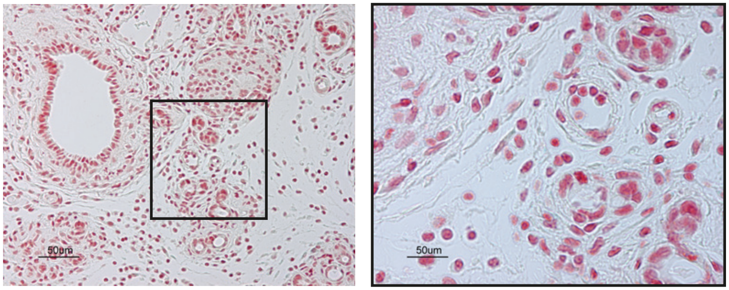

Supplementary Figure 1: No unspecific staining was observed in WT mice after PDL. Wild type (WT) littermate control mice (n=5) underwent PDL surgery as descibed in Figure 1. Tissues were harvested and processed for XGAL staining. Representative image of a WT pancreas (tail) stained for XGAL 14 days after PDL.

Supplementary FIGURE 1

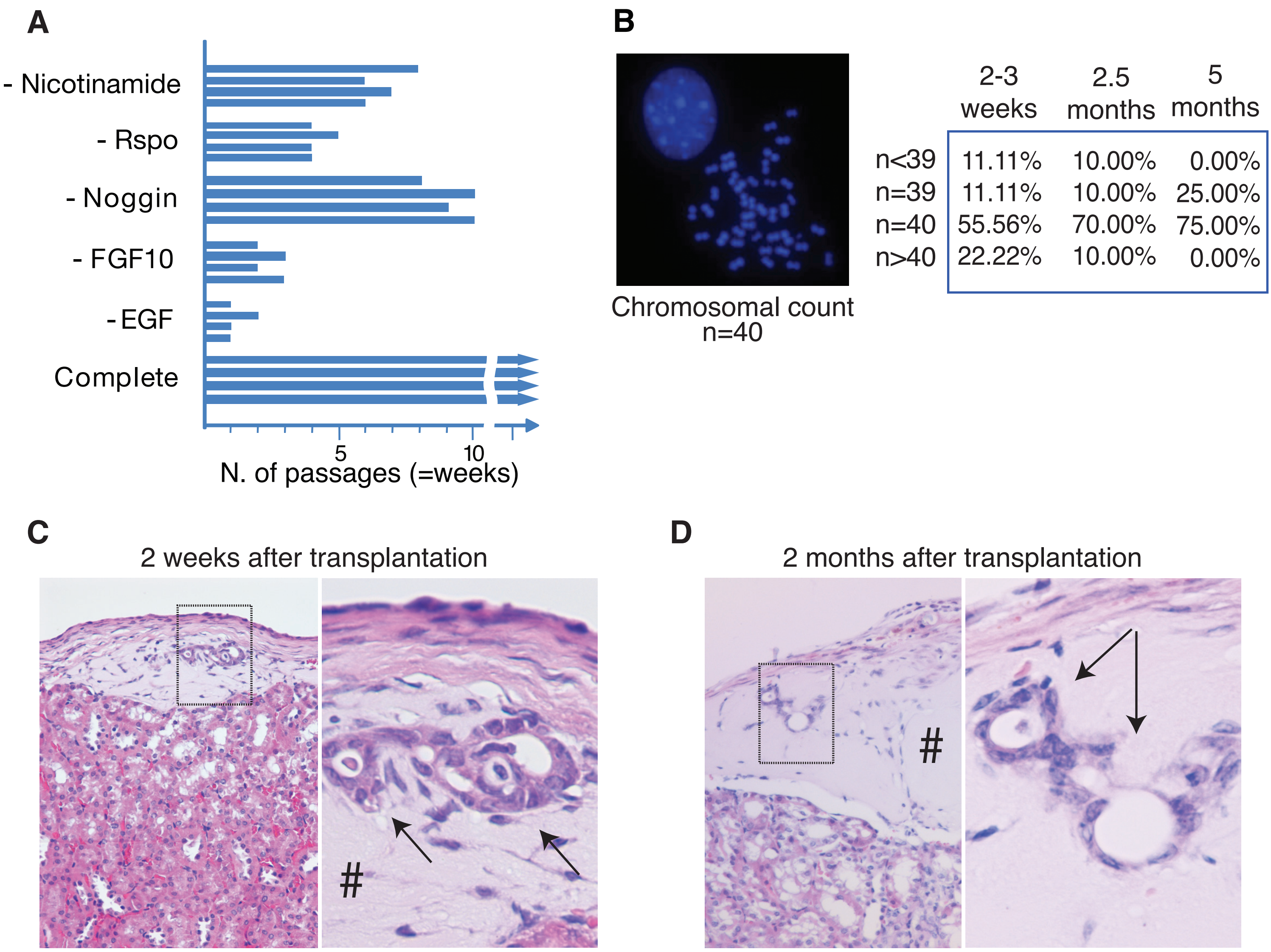

Supplementary Figure 2: Pancreatic organoid cells do not show any sign of transformation after long-term culturing. (A) Freshly isolated pancreatic duct derived organoids were cultured as described in Methods (complete) or in the absence of individual components, for the indicated time period. Each horizontal bar represents an independent experiment. Pancreatic organoids grow only when cultured with all the growth factors. **(B)** Genetic stability of the pancreas organoids cultured for long-term. Representative image of a chromosome spread illustrating a normal count (n=40) of a metaphase of a cell cultured for 175 days. The table illustrates the % of cells with chromosomal counts <39, =39, =40 and >40. **(C-D)** Representative H&E staining of a organoid-derived graft transplanted under the kidney capsule and harvested 2 weeks **(C)** and 2 months **(D)** later. Only ductal-like structures surrounded by ECM (extra cellular matrix) (#) are detected at both time points. No signs of transformation were observed in the transplanted ductal cells (arrows). # indicates the ECM formed around the engrafted pancreatic organoid derived cells.

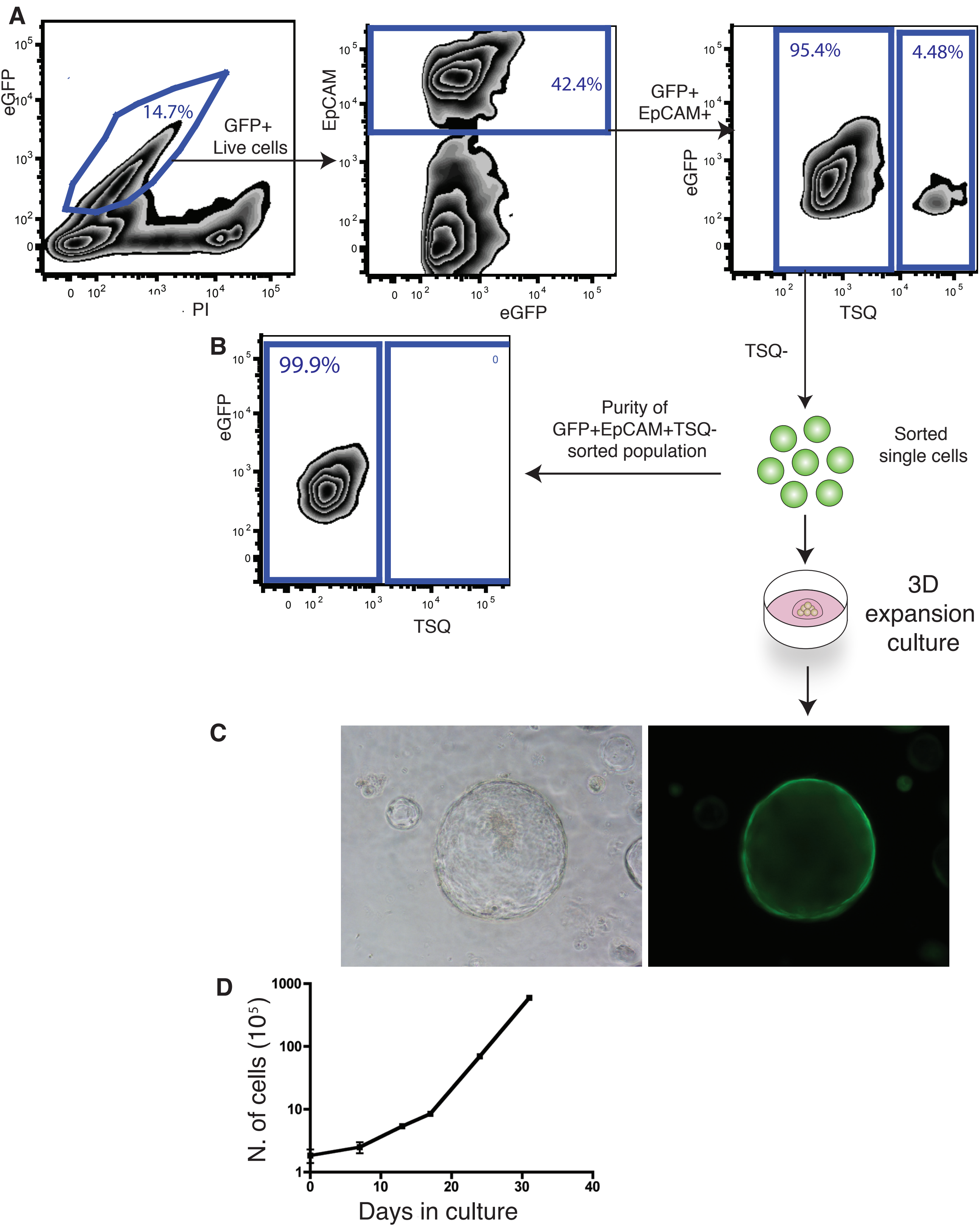

Supplementary Figure 3: Isolation and *in vitro* expansion of endocrine-depleted pancreatic epithelial cells from eGFP transgenic mice. (A) Fluorescence-activated cell sorting (FACS) plot illustrating the distribution of eGFP$^+$ live cells dissociated from an adult CAG^{eGFP+} mouse pancreas. eGFP$^+$ cells were sorted on the basis of EpCAM and TSQ expression. **(B)** EpCAM$^+$TSQ$^-$ cells were checked for purity (>99.9%) and plated in 3D expansion conditions. **(C)** eGFP$^+$ cells gave rise to expanding organoids as from Lgr5^{LacZ} and WT mouse pancreas cells. Magnification 10x. **(D)** Growth curve of pancreas organoids originated from EpCAM$^+$TSQ$^-$ sorted single cells cultured in our defined medium. Graph showing the number of cells counted per well at each passage. The cultures were expanded and split approximately once a week. Data represents mean ± S.E.M., n=2

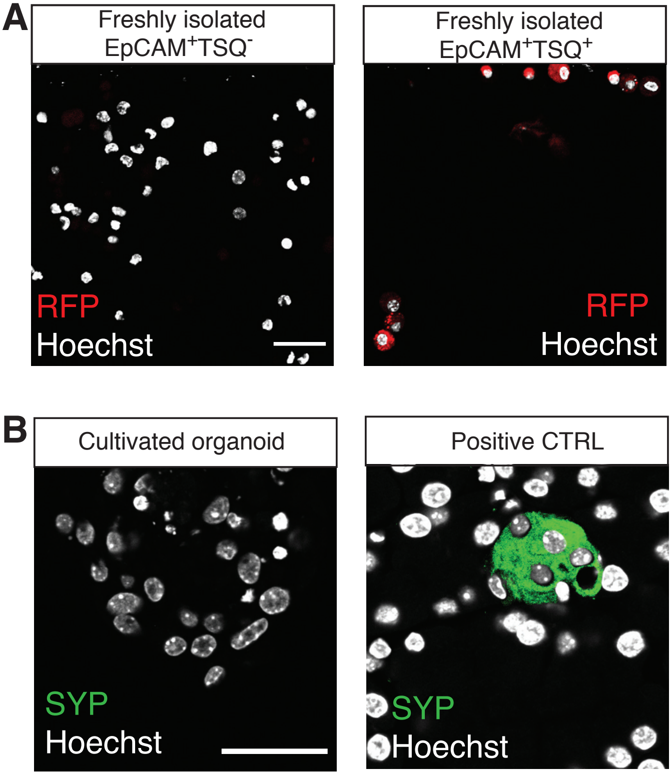

Supplementary Figure 4: Endocrine markers are not expressed in freshly isolated EpCAM^{+}TSQ^{-} cells and in expanding organoids.
(A) EpCAM^{+}TSQ^{-} and EpCAM^{+}TSQ^{+} sorted fractions from MipRFP mice were cytocentrifuged and direct RFP fluorescence was detected and imaged by confocal microscope (red); nuclei were counterstained with Hoechst 33342 (white). Scale bars = 20 µm. **(B)** Immunohistochemistry demonstrating absence of expression of the endocrine marker synaptophysin in an organoid growing for 6 passages *in vitro* (SYP, green). Positive control, adult pancreas tissue (SYP, green). Nuclei are counterstained with Hoechst 33342, white. Scale bars = 20 µm.

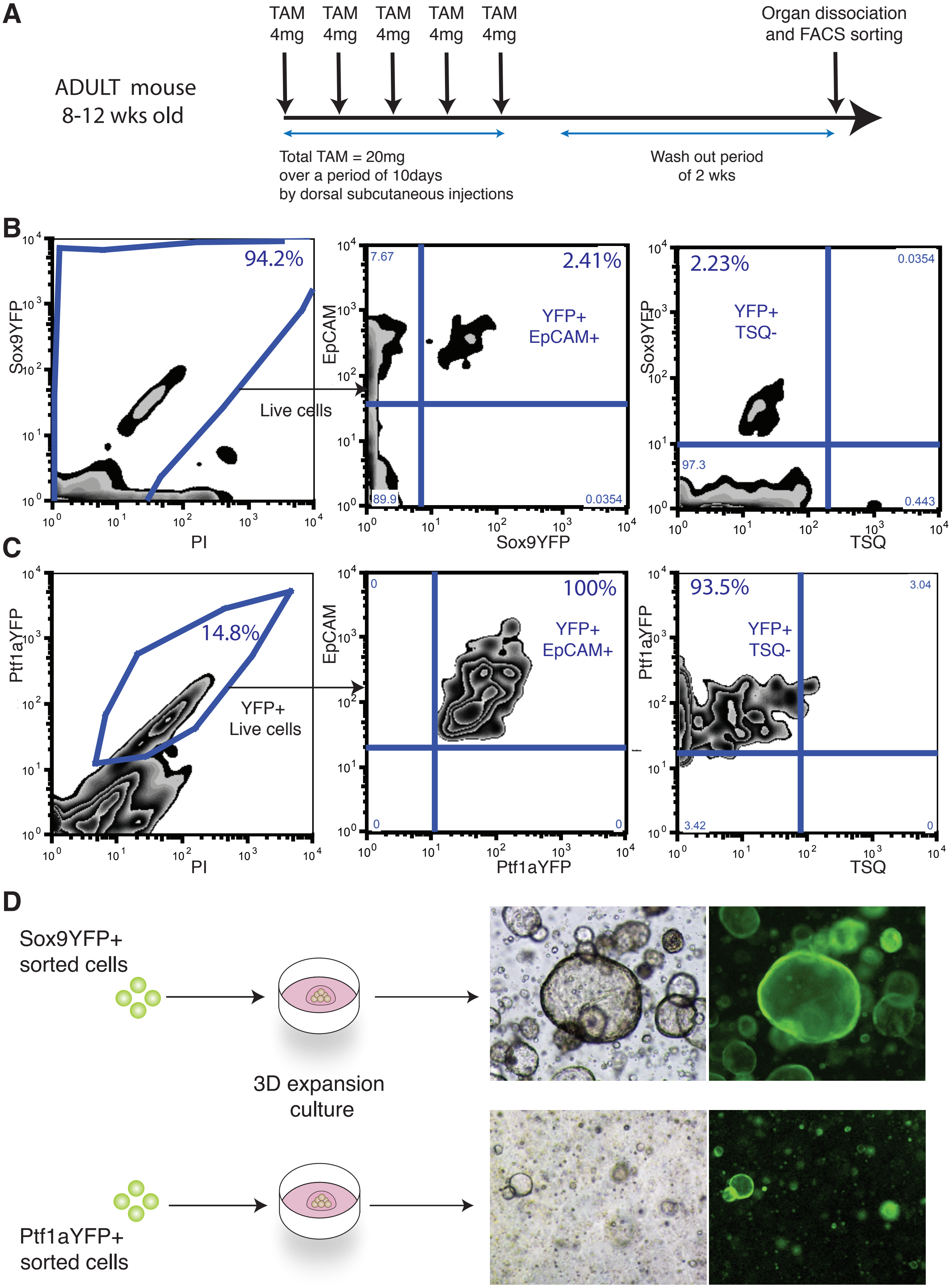

Supplementary Figure 5: Isolation and *in vitro* expansion of single, endocrine-depleted Sox9^{+} and Ptf1a^{+} pancreatic epithelial cells. (A) Schematic representation of Tamoxifen administration to Sox9^{CreER}xR26$^{fl/flYFP}$and Ptf1a^{CreER}xR26$^{fl/flYFP}$mice. Twenty milligrams of tamoxifen were injected subcutaneously over a period of 10 days. A wash out period of 2 weeks followed before pancreas harvesting and dissociation to single cells. **(B-C)** Fluorescence activated cell sorting (FACS) plot illustrating the distribution of YFP^{+}EpCAM^{+}TSQ^{-} cells derived from either Sox9^{CreER}xR26$^{fl/flYFP}$and Ptf1a^{CreER}xR26$^{fl/flYFP}$mice. **(D)** Schematic representation of cultures of YFP^{+} cells Sox9^{CreER}xR26$^{fl/flYFP}$and Ptf1a^{CreER}xR26$^{fl/flYFP}$. Images show that Sox9^{+} cells give rise to large duct-like structures that can be expanded for several weeks, while Ptf1a^{+} cells generate small duct-like structures that cannot be amplified for more than 3-4 passages. Magnification: 4x.

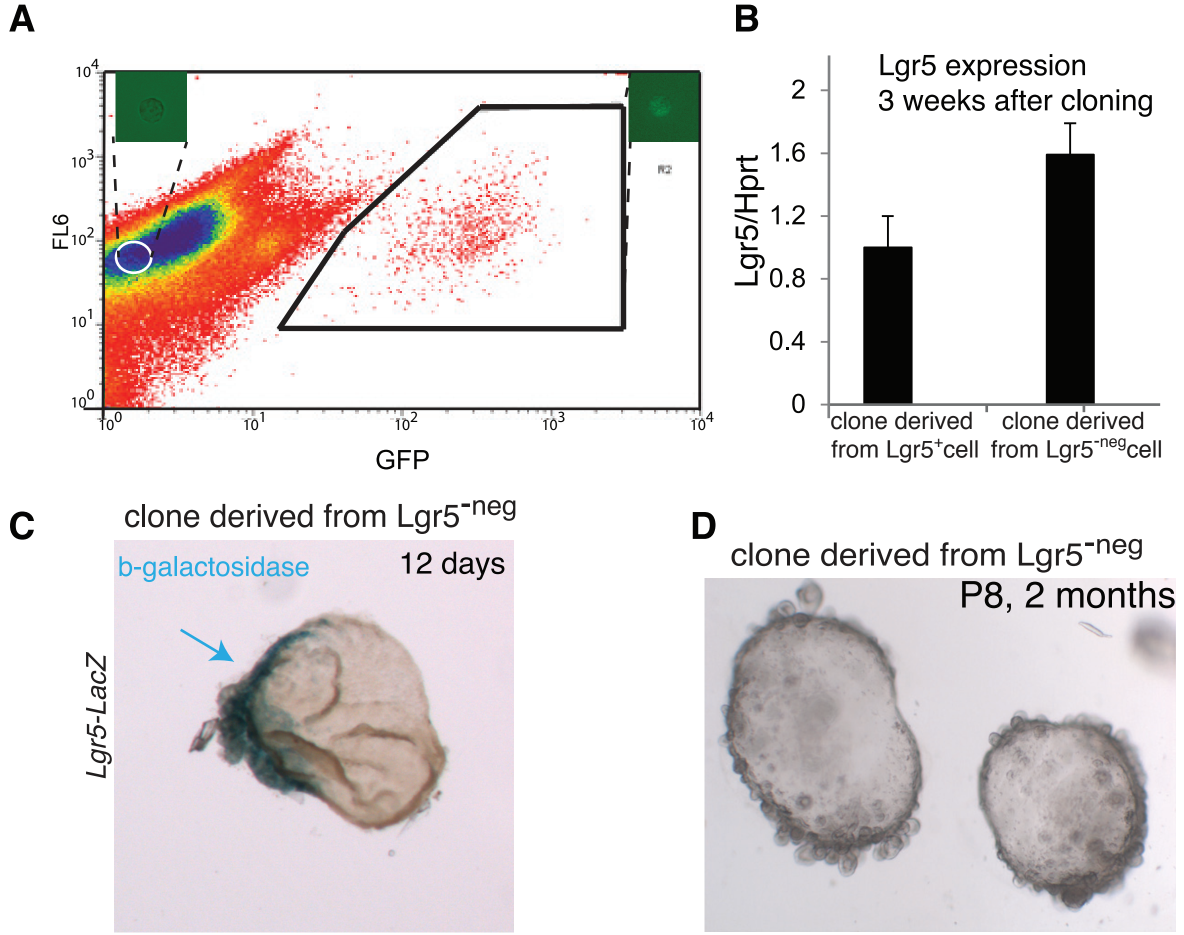

Supplementary Figure 6 *Lgr5* negative clones re-express *Lgr5* after culturing

Pancreas organoids derived from Lgr5^{LacZ} mice were grown for at least 2 weeks in culture before being processed for b-galactosidase FACS analysis as described in figure 4. (**A**) Representative fluorescence-activated cell sorting (FACS) plot illustrating the distribution of Lgr5^{+} cells from dissociated Lgr5^{LacZ} derived pancreas cultures stained with Detectagene Green CMFDG (to detect beta-galactosidase expression). Representative fluorescence images illustrate Lgr5 negative (left panel) and Lgr5 positive (right panel) cells after sorting. (**B-D**) Both, positive and negative Lgr5 sorted populations were seeded in matrigel and cultured in our defined culture medium. The colony formation efficiency of the positive (Lgr5^{+}) and negative (Lgr5^{-neg}) fractions is illustrated in Figure 4 (16.03%, Lgr5^{+} vs 1.6%, Lgr5^{neg}). Lgr5 expression was determined in clones started from both Lgr5^{+} and Lgr5^{neg} fractions at 12 days (**C**) and 3 weeks (**B**) after seeding. (**B**) Quantitative PCR showing relative fold change of *Lgr5* expression normalized by *Hprt* in organoids derived from *Lgr5* positive (Lgr5^{+}) and negative (Lgr5^{-neg}) single cell derived clones. At 3 weeks after seeding Lgr5 clones derived from the Lgr5^{-neg} population re-expressed Lgr5 at the same level as the Lgr5^{+} derived clones. Data represents mean ± S.E.M (n=2, independent cultures). (**C**) Representative DIC image of an Xgal stained organoid derived from a clone established from the Lgr5 negative fraction. B-galactosidase (blue) staining indicates that Lgr5 expression is re-activated as early as 12 days after seeding. Magnification 4x. (**D**) Clones started from the negative fraction were serially passaged for at least 2 months in a 1:4 split ratio. Magnification 4x.

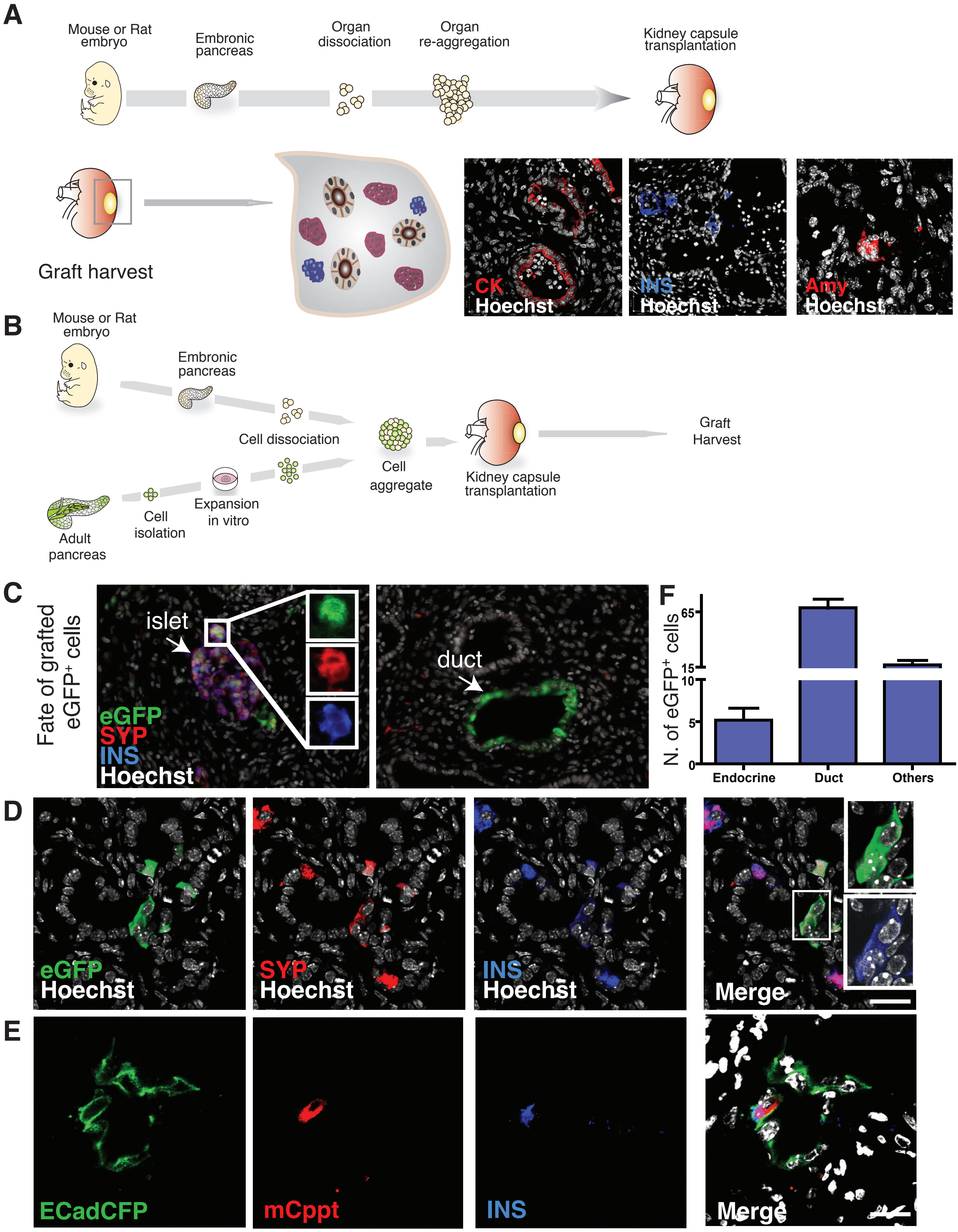

Supplementary Figure 7: *In vivo* morphogenetic assay for the pancreas. The developing organ facilitates the incorporation and differentiation of adult cultured pancreas organoid cells. **(A)** Pancreatic morphogenetic assay: embryonic mouse (E13) or rat (E14) pancreata are dissociated and then re-aggregated and grafted under the kidney capsule of nude mice. In the following 2 - 3 weeks time window the organ develops into all the 3 pancreatic lineages: duct cells (CK^+, red), endocrine cells (INS^+, blue) and acinar cells (AMY^+, red). Nuclei are counterstained with Hoechst (white). **(B)** Schematic representation of the morphogenetic assay where the embryonic pancreas is mixed with cultured organoid cells derived from either CAG^{eGFP+} or $ECad^{CFP}$ mice. Kidney capsule grafts were harvested and further processed for cryosection and immunohistochemistry. **(C)** $eGFP^+$ cells (green) were incorporated into both, duct (right panel) and endocrine (left panel) structures. Insets show higher magnification of $eGFP^+$ endocrine cells co-expressing both insulin (INS, blue) and synaptophysin (SYP, red) endocrine markers. **(D)** $eGFP^+$ cells derived from the cultured organoids differentiated into endocrine cells co-expressing both INS (blue) and SYP (red) endocrine markers. Insets show high magnifications of $eGFP^+INS^+$ cells. Magnifications: 20x. **(E)** $Ecad^{CFP+}$ mouse derived pancreas cultures were re-aggregated and grafted with rat embryonic pancreas (E14) as described. Representative confocal image of INS^+ (blue), mouse $Cppt^+$ (red) and $Ecad^{CFP+}$ mouse cells. All nuclei were counterstained with Hoechst 33342 (white, **C-E**). **(F)** Histogram showing the percentage ± S.E.M of endocrine (SYP^+), duct cells (CK^+) and other cell types (others) detected by double immuno-fluorescence in 11 grafts from 6 independent cultures.

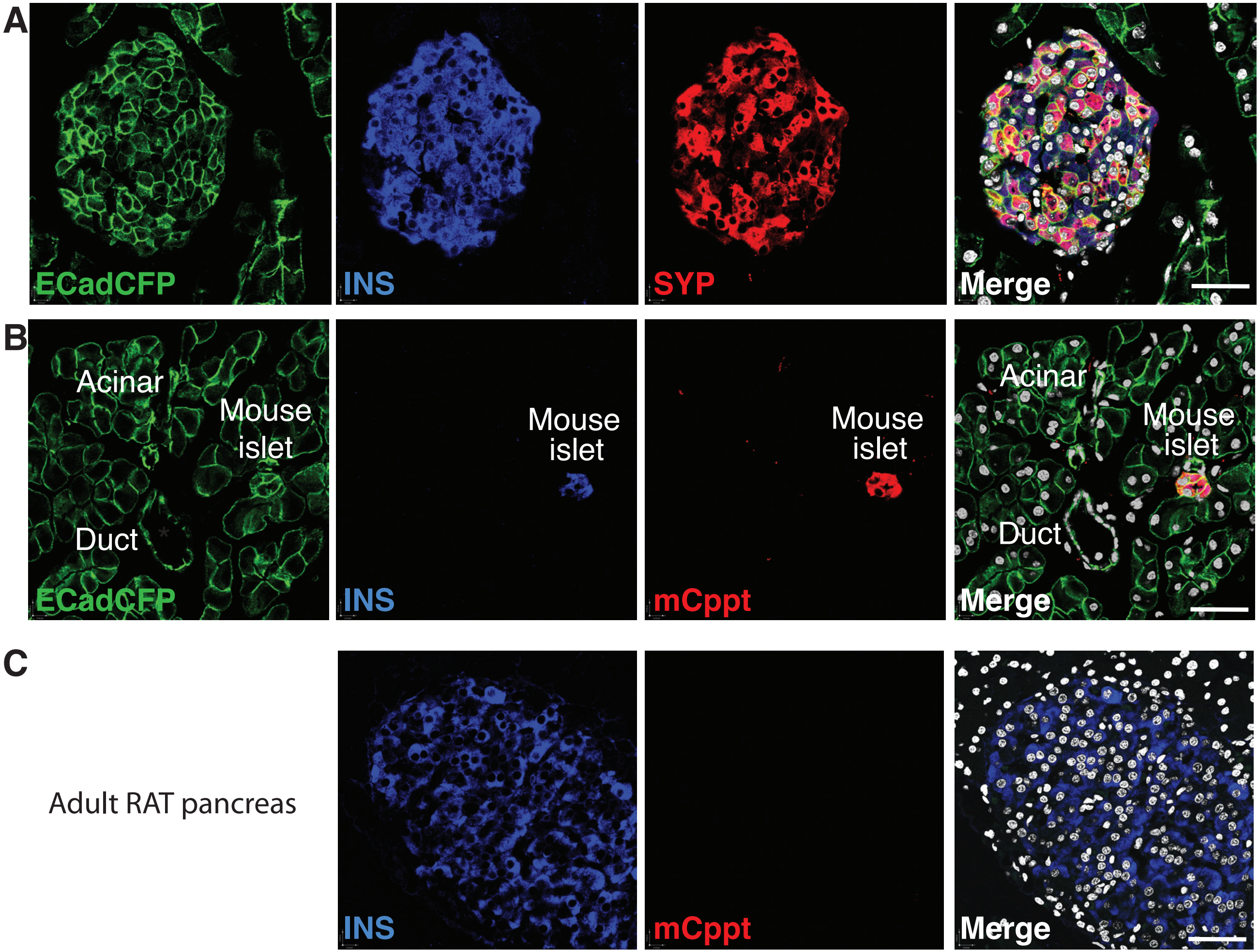

Supplementary Figure 8: Characterization of ECad^{CFP} mouse pancreas and assessment of mouse specificity of anti-Cppt antibody. (A) Confocal microscopy images illustrating the membrane localization of CFP (green) in the adult mouse pancreas of ECad^{CFP} mice. Note that Endocrine INS^{+} (blue) and SYP^{+} (red) cells are all positive for CFP. Scale bar: 35µm **(B)** INS^{+} (blue) cells of the adult mouse pancreas stain positive for the mouse specific anti-Cppt while INS^{+} (blue) cells of the adult rat pancreas **(C)** are negative for the mouse Cppt staining, demonstrating species-specificity of the anti-Cppt antibody. Scale bar: 35µm **(B)** and scale bar: 40µm **(C)**.

Supplementary FIGURE 8

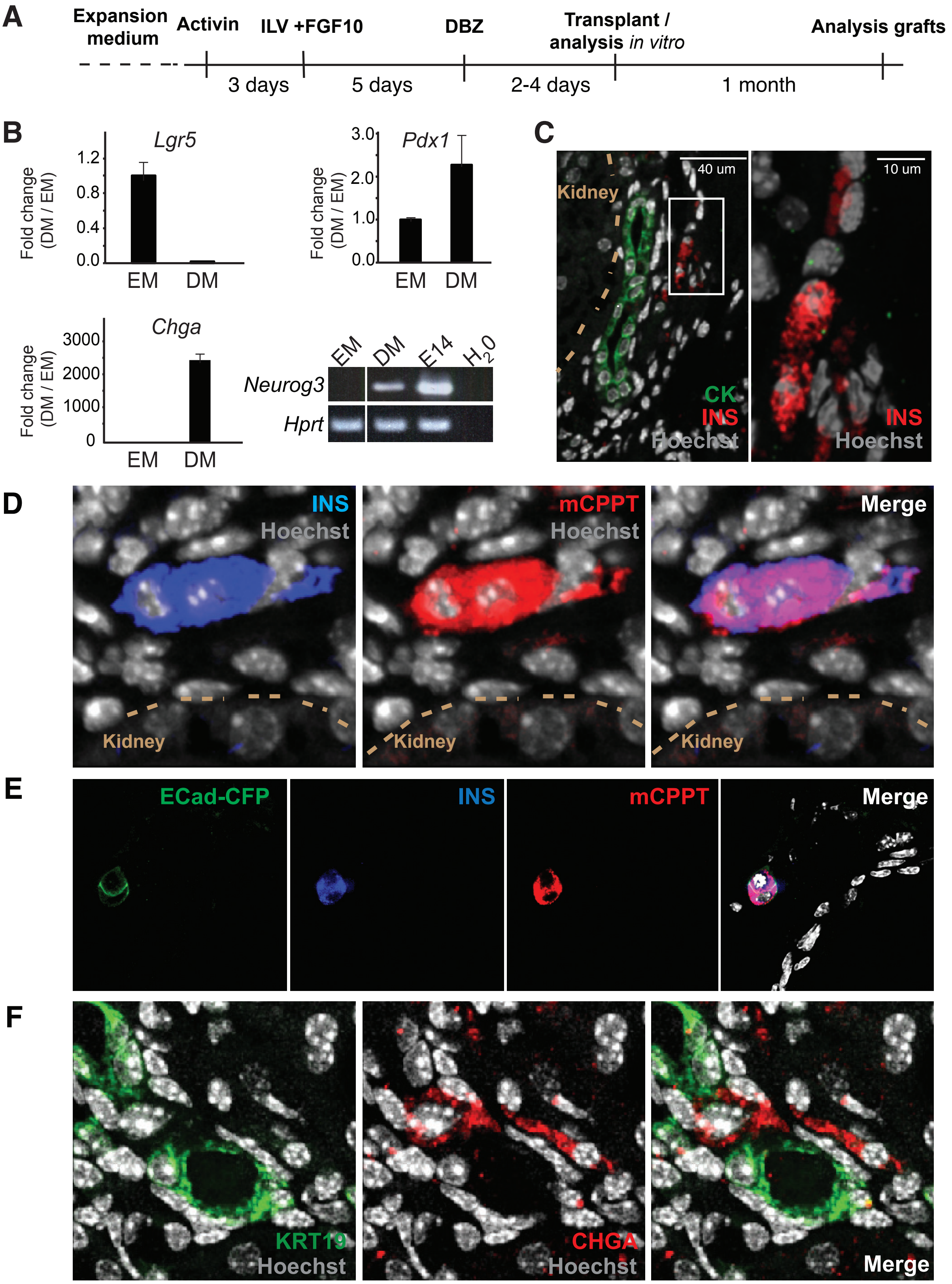

Supplementary Figure 9 Direct transplantation of pancreas organoid cultures into the kidney capsule of nude mice (**A**) Differentiation protocol used to "prime" the organoids to an endocrine fate prior to transplantation. The detailed protocol is described in Material and Methods. ILV, indolactam-V; FGF10, fibroblast growth factor 10. (**B**) Quantitative PCR showing relative fold changes of *Lgr5*, *Pdx1* and *Chga* mRNA levels in pancreas organoids cultured in our defined expansion medium (EM) or after being treated with the differentiation protocol (DM) as described in (A). Results were normalized to *Hprt*. Results are expressed as mean ± SEM of 2 independent experiments. *Neurog3* was detected by semi-quantitative RT-PCR. *Hprt* was used as internal reference. Embryonic E14 pancreas RNA was used as + control. Two independent experiements were performed. Representative image is shown. (**C-F**) Organoids derived from Bl6 mouse (**C-D, F**) or ECad^{CFP} mouse (**E**) expanded for at least 2 months in culture were differentiated as described in (A), and transplanted directly into the kidney capsule of nude mice. One month after transplantation, the kidneys were collected and the grafts analyzed for the presence of duct and endocrine cells from the donor organoid cells. (**C**) Pancreas organoids readily generated duct-like structures, as detected by CK (pan-cytokeratin, green) staining. Some cells delaminated from the ducts and differentiated towards endocrine, INS (insulin, red) positive, cells. (**D**) Representative confocal image of organoid derived cells expressing both INS (blue) and mCPPT (mouse specific C-peptide, red) in a 1 month-old graft. (**E**) Representative confocal image of transplanted ECad-^{CFP} positive cells co-expressing INS (blue) and mCPPT (red). (**F**) KRT19 (keratin 19, green) duct-like structures surrounded by endocrine committed cells expressing CHGA (Chromogranin A, red) were detected 1 month after transplantation. Representative confocal image is shown. In all cases, Nuclei were stained with Hoechst. Magnifications 40x (**E**) and 63x (**D,F**).

SUPPLEMENTARY TABLE I

Antibody	Antibody clone/ reference	Raised	Dilution	Origin
PanCytokeratin (PCK)	Z0622	rabbit	1:500	Dako
Insulin [1]	046K4853	mouse	1:8000	Sigma
Pdx1	polyclonal	Guinea Pig	1:10.000	BCBC (C. Wright)
Ki67	Clone TEC-3 / M7249	Rat (IgG2a)	1:1000	Dako
Sox9	AB5535	rabbit	1:600	Millipore
MIC1-1C3	NBP1-18961C	rat (IgG2a)	1:200	Novus biological
Mucin-1	Clone Ab-5	Armenian hamster	1:100	Thermo Scientific
a-Amylase	Polyclonal / A8273	Rabbit	1:500	Sigma
Krt19[2]	M 0888	Mouse IGg1	1:20	Dako
Chromogranin A (ChrA)	Sc1488 (C-20)	Goat	1:100	Santa Cruz
Synaptophysin	Polyclonal / Z66	Rabbit	1:500	Invitrogen
EpCAM -APC	Clone G8.8 / 17-5791	Rat IGg2a	1:350	eBiosciences
Insulin	AB3440	Guinea Pig	1:250	Millipore
Mouse C-peptide	Polyclonal/ AB1044	Rabbit	1:1500	BCBC
Glucagon	Polyclonal	Rabbit	1:3000	Diabetes Reaserch Center
Somatostatin	SKU# 18-0078	Rabbit	1:1000	Invitrogen
Krt19[3]	Troma III	rat	1:200	Hybridoma bank

[1] Used in paraffin sections (Figure 1)
[2] Used in figure 5
[3] Used in supplementary fig 9

SUPPLEMENTARY TABLE II

Gene name	Gene Symbol		Sequence primers used in Fig1 and Suppl. Fig 9	PCR product (bp)
SRY-box containing gene 9	*Sox9*	fw	CTCCTAATGCTATCTTCAAG	200
		rv	GCTTCAGATCAACTTTGC	
Pancreatic and duodenal homeobox 1	*Pdx1*	fw	CCCGGACCTTTCCCGAATGG	144
		rv	CTCGGGTTCCGCTGTGTAAGC	
Neurogenin 3	*Neurog3*	fw	CGCACCATGGCGCCTCATCCCTTGG	664
		rv	CAGAGGATCCTCTTCACAAGAAGTCTGAG	
Chromogranin A	*Chga*	fw	CTCGTCCACTCTTTCCGCAC	176
		rv	CTGGGTTTGGACAGCGAGTC	
Leucine-rich repeat-containing G-protein coupled receptor 5	*Lgr5*	fw	GGAAATGCTTTGACACACATTC	450
		rv	GGAAGTCATCAAGGTTATTATAA	
Axin 2	*Axin2*	fw	TGTCCAGCAAAACTCTTC	120
		rv	CTTCTCTTGAAGGACCTGA	
Insulin 2	*Ins2*	fw	CCATCAGCAAGCAGGAAG	188
		rv	GGGTGTGTAGAAGAAGCC	
Hypoxanthine phosphoribosyltransferase	*Hprt*	fw	AAGTTTGTTGTTGGATATGC	106
		rv	CATCTTAGGCTTTGTATTTGG	

Gene name	Gene Symbol	Primers and probe sets used in qPCR – IDT (Figure 5)
Leucine-rich repeat-containing G-protein coupled receptor 5	*Lgr5*	Probe: 5'-/56-FAM/TGAGAAGCC/ZEN/TTCAATCCCTGCGC/3IABkFQ/-3'<br>Primer fw: 5'-ACGTAGCTGATGTGGTTGG-3'<br>PrImer rv: 5'-GCCTCAAAGTGCTTATGCTG-3'
pancreatic and duodenal homeobox 1	*Pdx1*	Probe: 5'-/56-FAM/AGGAGGTGC/ZEN/TTACACAGCGGAAC/3IABkFQ/-3'<br>Primer fw: 5'-GCAGTACGGGTCCTCTTGT-3'<br>PrImer rv: 5'-GATGAAATCCACCAAAGCTCAC-3'
SRY-box containing gene 9	*Sox9*	Probe: 5'-/56-FAM/CCCTGAGAT/ZEN/TGCCCAGAGTGCTC/3IABkFQ/-3'<br>Primer fw: 5'-CTCGCTTCAGATCAACTTTGC-3'<br>PrImer rv: 5'-ACTCCCCACATTCCTCCT-3'
Amylase 2a5	*Amy2a5*	Probe: 5'-/56-FAM/ACTGGCCTT/ZEN/CTGGATCTTGCACTT/3IABkFQ/-3'<br>Primer fw: 5'-ACTCTGCTTGGGACTTTAACG-3'<br>PrImer rv: 5'-CACCTTGGTACGAACATAATCTTTC-3'
Insulin I	*Ins1*	Probe: 5'-/56-FAM/CATCCAGTA/ZEN/ACCCCCAGCCCTTAG/3IABkFQ/-3'<br>Primer fw: 5'-GCCATGTTGAAACAATGACCTG-3'<br>PrImer rv: 5'-GCCAAACAGCAAAGTCCAG-3'
Cyclophilin A	*CyA*	Probe: 5'-/5TET/TCCGTA/ZEN/GGACCTGCCGC/3IABkFQ/-3'<br>Primer fw: 5'-TTCCAGGATTCATGTGCCAG-3'<br>PrImer rv: 5'-CTGGGAACCGTTTGTGTTTG-3'
